# Factors affecting effectiveness of food control inspections in food production establishments in Finland

**DOI:** 10.1038/s41598-022-08204-1

**Published:** 2022-03-10

**Authors:** Mikko Kosola, Katri Kiviniemi, Janne Lundén

**Affiliations:** Department of Food Hygiene and Environmental Health, Faculty of Veterinary Medicine, University of Helsinki, P.O. Box 66, 00014 Helsinki, Finland

**Keywords:** Gastrointestinal diseases, Disease prevention

## Abstract

Inspections are an important tool for food control. However, there is a lack of knowledge about how inspection history, interval between inspections, and pre-announcement of the inspection affect compliance with food safety legislation in food production establishments. We used register-based meat, fish, and dairy establishment food control inspection data (5550 inspections from 757 establishments) from Finland in 2016–2019 to study compliance in relation to these factors. Hypothesis was that there is an association between inspection grades and (1) inspection history, (2) interval, and (3) pre-announcement. Results indicate that minor non-compliances that do not impair food safety often precede future more severe non-compliances (5.3% non-compliance rate if full compliance at previous inspection compared to 16.8% non-compliance rate if minor non-compliances at previous inspection [Fisher’s exact test, *p* < 0.0001]), and that longer inspection intervals are associated with a decrease in inspection grades (5.9% difference in inspection intervals for inspection with full compliance compared to inspections with severe non-compliances [Generalized estimating equations, *p* = 0.02]). In addition, pre-announcement of the inspection affects the inspection grades, severe non-compliances were 2.4 times more common at unannounced inspections compared to pre-announced inspections. To conclude, there is an association between inspection grades and inspection history, inspection interval, and inspection announcement.

## Introduction

The prevention of foodborne illnesses is the most important objective of food control. The foundation for food control in the European Union stems from EU regulations EC 178/2002^1^ and EC 2017/625^2^, and is supplemented in Finland by national legislation^3^. These regulations set concordant principles for food control throughout the entire food chain. Inspections are an important tool for food control. One of the major objectives of food control is the endeavor for risk-based inspections^2^. In Finland, the Finnish Food Authority has provided guidelines, based on the type and output of production, for the evaluation of the risk levels of food production establishments^4^. The risk level determines the frequency of inspections at the respective establishment. These guidelines also state that the inspection history can have an effect on inspection frequency; an excellent inspection history can lead to a 50% decrease in inspection frequency. However, the effect of inspection history on inspection grades has not been conclusively established. In restaurants in Finland, the proportion of inspections with major non-compliances has been found to be significantly higher if minor non-compliances were found during a previous inspection compared to restaurants that were fully compliant^5^. However, to our knowledge the effect of inspection history on inspection grades at food production establishments has not been studied in Finland or elsewhere. By increasing knowledge about the effects of inspection frequency on compliance, more sophisticated models for the determination of inspection frequency can be used when future food control guidelines are established.

The association between inspection frequency and inspection results has been studied with conflicting results. Bader et al.^6^ found that a decrease in inspection frequency from four times to once a year impaired the inspection result. Allwood et al.^7^ studied restaurants that had different inspection frequencies during two consecutive years and noticed that inspection scores deteriorated when the inspection frequency decreased. Leinwand et al.^8^ found that an increase in inspection frequency decreased violations in nonchain restaurants, but not in chain restaurants. On the other hand, some studies have not found an association between inspection frequency and inspection results^9,10^. All of these studies have focused on restaurant inspections. To our knowledge, there are no studies that have focused on food production establishments. Food production establishments manufacture food products before the retail stage. Their products are often distributed widely to food stores and restaurants and thus potential food safety problems could affect a large group of consumers.

In Finland, only a few studies have touched upon inspection intervals. Läikkö-Roto et al.^11^ found that inspectors increased the inspection frequency if food business operators (FBOs) failed to execute corrective actions. Another study examined the perceptions of FBOs toward food control^12^. The authors reported that the frequency of inspections correlated positively with FBOs’ perceptions of the relevance of non-compliances to food safety and the attitudes of FBOs toward food control. In a recent study, an association between inspection interval and inspection grades was not demonstrated in restaurants^5^.

EC 2017/625^2^ states that inspections should be performed unannounced, unless pre-announcement is necessary for the inspection to be carried out. Unannounced inspections are important as they reflect the true situation of the establishment. However, pre-announced inspections have also been found to have positive effects as they support active managerial control that can yield improved inspection grades in forthcoming inspections^13^. It is important to ascertain the effect of pre-announcement on inspection grades because this knowledge can be useful in the planning of inspections. Pre-announcement of an inspection has been shown to affect inspection grades^5,14^. Kaskela et al.^5^ found that the proportion of unsatisfactory inspection grades was at least two-fold in most of the inspected items in unannounced inspections compared to pre-announced inspections, and Waters et al.^14^ noticed this in part of the inspection categories. Differences were seen in both studies, especially regarding items that are easy to correct. The effect of pre-announcements on inspection grades in food production establishments has not been studied earlier.

The purpose of this study was to investigate the association between (1) previous inspection results, (2) inspection interval, and (3) inspection pre-announcement and inspection grades at meat, fish, and dairy establishments. These objectives have earlier been studied only in a restaurant setting but not in food production establishments. Our hypothesis was that (1) inspection history predicts inspection results, (2) longer inspection intervals lead to inferior inspection results, and (3) pre-announcement of the inspection allows FBOs to react before the inspection, which results in better grades not reflecting the true situation in the establishment.

## Materials and methods

### Establishments and inspections

The materials for this study included food control inspection reports from all inspected meat, fish, and dairy establishments from 2016 to 2019 in Finland. Large-scale slaughterhouses were excluded because the inspection protocol for them differs from other establishments. In large-scale slaughterhouses, inspections are conducted continuously but reported only at pre-defined intervals^15^, whereas in other establishments inspections are performed with a pre-defined frequency and a report is compiled after each inspection. Altogether, there were 757 establishments and 5550 inspections in the data (Table 1). Inspections have been carried out by local food control inspectors, and data was provided by the Finnish Food Authority that is the central agency responsible for directing food control in Finland.

**Table 1:** Descriptive statistics of food control inspections of meat, fish, and dairy establishments in Finland 2016–2019.

		Establishment type			
Parameter	Total	Meat	Fish	Dairy	Mixed
Establishments (*n*)	757	283	332	126	16
Establishments with at least one pre-planned* inspection (*n*)	639	236	282	105	16
Inspections (*n*)	5550	2481	1913	948	208
Mean inspection interval of pre-planned* inspections in days (SD)	137.2 (132.5)	113.0 (110.8)	179.4 (160.2)	127.4 (107.7)	94.3 (113.8)
Number of pre-planned* inspections	4004	1741	1335	768	160
Overall grades (%) of pre-planned* inspections					
Excellent (A)	47.1	40.8	45.6	66.4	34.4
Good (B)	44.0	47.4	46.0	30.6	54.4
To be corrected (C)	8.7	11.3	8.2	3.0	11.3
Poor (D)	0.3	0.5	0.2	0	0
Pre-announced pre-planned* inspections (*n*)	2929	1147	1003	670	109
Overall grades of pre-announced pre-planned inspections (%)**					
Excellent (A)	50.6	45.2	47.9	66.1	38.5
Good (B)	42.7	46.0	45.7	31.2	51.4
To be corrected (C)	6.5	8.5	6.4	2.7	10.1
Poor (D)	0.2	0.4	0.1	0	0
Unannounced pre-planned* inspections*(n)*	828	403	295	91	39
Overall grades of unannounced pre-planned inspections (%)**					
Excellent (A)	37.8	33.0	38.3	68.1	12.8
Good (B)	45.8	46.9	46.4	26.4	74.4
To be corrected (C)	16.2	19.6	15.3	5.5	12.8
Poor (D)	0.2	0.5	0	0	0

*Defined as (1) having overall grade A or B at the previous inspection, (2) marked as pre-planned inspection, and (3) having at least 14 days inspection interval.

**247 inspections for which information about pre-announcement was missing.

The Finnish food control system includes 97 individual inspection items for food production establishments. However, only some of the items are inspected at each visit. Each inspected item is given a grade on a four-level scale: A = excellent, complies with requirements; B = good, minor issues that do not impair food safety or mislead consumers; C = to be corrected, issues that impair food safety or mislead consumers; D = poor, major issues that considerably impair food safety or mislead consumers, or the FBO has failed to correct non-compliances^16^. Up-to-date evaluation guidelines of inspection items are available at the website of the Finnish Food Authority^17^. The overall inspection grade is determined by the lowest individual item grade. Overall grades C and D lead to a follow-up inspection in which the correction of observed non-compliance is assessed. The frequency of other inspections is pre-planned based on the risk level of the FBO.

The whole data were used when we examined the changes in inspection-item-specific grades between consecutive inspections. When investigating the effect of pre-announcement on inspection grades and the effect of the previous inspection grade on the grade of the subsequent inspection, only pre-planned inspections were included in the analyses. In our analyses we defined an inspection as pre-planned if (1) the overall grade of the previous inspection was A or B, and (2) the inspection was marked as pre-planned. Inspections marked as pre-planned which had overall grade C or D at the previous inspection were not included because it is common practice that if the inspection interval for a pre-planned inspection is suitable, a follow-up inspection of a non-compliance is carried out during the pre-planned inspection. In addition, as the number of pre-planned inspections at the highest risk-level establishments is 12 inspections per year, corresponding to one per month, we excluded inspections with an inspection interval of less than 14 days from these analyses because a short interval indicates that inspections are not truly two consecutive pre-planned inspections. There were 4004 pre-planned inspections altogether, out of which 2929 were pre-announced and 828 were unannounced (Table 1). Information regarding pre-announcement was missing from 247 pre-planned inspections.

Analyses concerning the association between inspection interval and inspection grades were also conducted for pre-planned inspections. For these analyses, our statistical methodology required the establishment to have at least two pre-planned inspections. Thus, only 520 establishments with 3492 inspections were included. We did not study the impact of inspection interval on follow-up inspection grades. This is because the inspection interval is dependent upon the severity of the non-compliance and the time limit set to correct it, and we lacked information on the time limit for correction.

### Statistical analysis

As inspection frequencies for establishments differ greatly, absolute inspection intervals are not comparable. In order to make inspection intervals between establishments comparable, we standardized them by calculating standard scores. Standard scores were calculated as the difference between the inspection interval and the mean inspection interval of the establishment, divided by the standard deviation of inspection intervals of that establishment^18^. A standard score of -0.5 means that the inspection was carried out half a standard deviation earlier than on average for that establishment while, conversely, 0.5 means that the inspection was carried out half a standard deviation later than on average.

To account for the correlation caused by the non-independence of observations, we used generalized estimating equations (GEE)^19^ with an identity link function and exchangeable working correlation structure to analyze the association between inspection interval and inspection grades. The standard score was used as a dependent variable representing length of inspection interval. The independent variable of interest was the inspection grade. The grade of the previous inspection was controlled as only pre-planned inspections were included in the analyses. To capture the inspection history further, analyses were adjusted for the grade of the second to last inspection. “Establishment” was incorporated into the analyses as a repeated term. The interaction between the overall inspection grade and the overall grade of the previous inspection was examined and if an interaction was found, the analyses were interpreted separately in the different classes based on the previous overall grade. As there was a very low number of inspections that resulted in a grade D, grades C and D were combined in the analyses. The GEE approach was also used when studying the effect of the inspection interval on individual inspection items. In these analyses, the inspection interval was calculated from the inspection at which the item in question was previously inspected. Only inspection items that had at least five C or D grades were included to avoid any misconceptions caused by infrequent observations. The results are presented as mean standard score estimates for inspections resulting in grades A, B or C/D and as the p-value comparing their differences. To make the results more accessible, the average difference in inspection intervals is also presented as a percentage.

**Figure 1:**
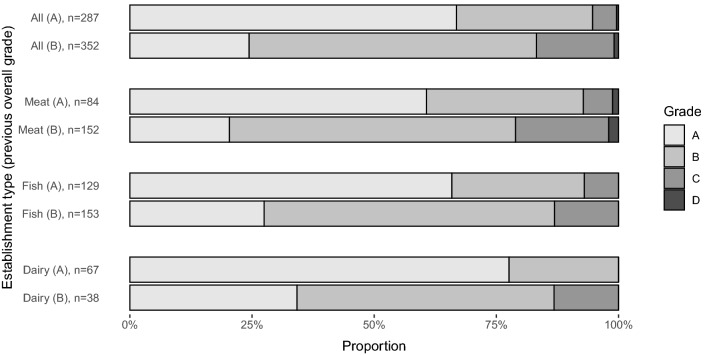
Grade distribution of the food production establishments’ first pre-planned inspection grouped by the grade of the previous inspection. Grade of the previous inspection denoted in parenthesis; A = Excellent, B = Good.

To study the differences in grade distributions between inspections that had an overall grade of A or B at the previous inspections, Fisher’s exact test^20^ was used. To avoid the problem of the non-independence of observations, only the first pre-planned observation from each establishment was included in the analyses. To study the association between the overall inspection grade (converted to a numerical variable: A = 1, B = 2, C = 3, and D = 4) and the number of inspected items graded B at the previous inspection (if the overall grade of the previous inspection was B), the Spearman correlation of subject means was used. All analyses were conducted using SAS 9.4 (SAS Institute Inc., Cary, NC, USA). Figure 1 was created with R version 4.0.0 (R Core Team, 2020). Statistical significance was determined as p < 0.05.

## Results

### Distribution and changes in overall inspection grades

Of the pre-planned inspections, 47.1% were graded A, 44.0% were graded B, 8.7% were graded C, and 0.3% received grade D (Table 1). There were marked differences between establishment types: 66.4% of inspections at dairy establishments resulted in grade A, whereas at meat establishments, the respective number was 40.8%. Differences were also seen in the proportions of grades C and D. At dairy establishments, only 3.0% of inspections received grade C or D, whereas in meat establishments this figure was 11.8%.

The distribution of overall grades of the first pre-planned inspections in the study period differed between those establishments in which the overall grade of the previous inspection had been A compared to those in which the grade had been B (Fig. 1). Changes from grade A to grade C or D occurred in only 5.3% of the inspections, whereas changes from grade B to grade C or D occurred in 16.8% (Fisher’s exact test, *p* < 0.0001). This difference was also seen separately in meat, fish, and dairy establishments (Fisher’s exact test; *p* < 0.0001) (Fig. 1). When looking at inspections for which the overall grade of the previous inspection was B, the number of inspection items graded B at the previous inspection correlated with the overall inspection grade of the next inspection (converted to a numerical variable), meaning that inferior overall grades were associated with more inspection items graded B at the previous inspection (Spearman correlation coefficient 0.43, *p* < 0.0001).

### Changes in inspection-item-specific grades

When looking at individual inspection items (Supplementary Table S1), changes in grades between inspections from grade A to grade C or D were infrequent (range 0–3.7%). The most substantial change in compliance was seen in “General labeling”, in which 3.7% of inspections graded A plummeted to C or D at the next inspection. Changes from grade B to grade C or D were more frequent (range 1.9–13.5%). There were five inspection items in which there was a 10% or higher proportion of grade B declining to C or D (“Approval of facilities, structures and equipment”, “General labeling”, “Sampling and own-check tests”, “Own-check testing of water and ice”, and “Own-check of listeria”). When looking at corrections of non-compliances, there were 10 inspection items in which grade C or D had not been corrected in over 30% of inspections ("General compliance of own-check with requirements”, “Maintenance of facilities and structures”, “Cleanliness and order of facilities and structures”, ”Cleanliness of surfaces, fixtures, equipment and utensils”, ”Vermin control”, ”Temperature management in chilled facilities”, “Traceability of foodstuffs”, ”Sampling and own-check tests”, ”Own-check of listeria”, and ”Display of the inspection report”). For example, non-compliances leading to grade C or D were not corrected in 48.9% of follow-up inspections in the case of the inspection item “Sampling and own-check tests”. The corresponding figure was 47.1% for “General compliance of own-check with requirements”, 38.5% for “Traceability of foodstuffs”, and 36.2% for “Maintenance of facilities and structures”.

### Effect of announcement on inspection grades

For announced inspections, 50.6% received grade A, whereas for unannounced inspections, the corresponding figure was 37.8% (Table 1). Further, 6.5% of pre-announced and 16.2% of unannounced inspections received grade C. When looking at establishment types separately, the results were similar in meat and fish establishments. However, in dairy establishments, grade A was more common in unannounced inspections (68.1%) compared to announced inspections (66.1%). There were 14/15 inspection items for which grades C and D were at least three times more common for unannounced inspections than for pre-announced inspections (Table 2). The largest differences between pre-announced and unannounced inspections were seen in the inspection items “Display of inspection report” (11.7x), “Hygiene in storage and warehousing of foodstuffs” (9.2x), and “Working clothes and protective clothing of personnel” (7.8x).

**Table 2:** Grade distribution of inspection items on unannounced (1st line) and pre-announced (2nd line) pre-planned inspections.

Inspection item	N**	Grade*				Quotient of the proportion of C and D grades (unannounced/announced)
		A	B	C	D	
Display of the inspection report	382	89.5	9.4	1.1	0	11.7 ×
	1063	93.0	6.9	0.1	0	
Hygiene in storage and warehousing of foodstuffs	414	75.9	19.1	4.8	0.2	9.2 ×
	1281	88.9	10.5	0.6	0	
Working clothes and protective clothing of personnel	532	89.7	9.4	0.9	0	7.8 ×
	1611	95.9	4.0	0.1	0	
Working hygiene of personnel	498	84.1	12.5	3.4	0	7.1 ×
	1449	92.3	7.2	0.5	0	
Separation of activities requiring different hygiene levels	273	78.0	19.1	2.9	0	6.1 ×
	1039	89.1	10.4	0.5	0	
Hygiene in wrapping and packing	241	86.7	11.2	2.1	0	5.9 ×
	861	93.2	6.5	0.2	0.1	
Cleanliness of surfaces, fixtures, equipment and utensils	632	80.5	17.4	2.1	0	4.9 ×
	2162	90.9	8.7	0.4	0	
Cleanliness and order of facilities and structures	700	70.0	26.4	3.6	0	4.5 ×
	2353	86.7	12.5	0.8	0	
General hygiene of food production	235	79.2	17.9	3.0	0	4.3 ×
	1002	92.1	7.2	0.7	0	
Hygiene of water supply points and equipment using water	398	84.2	14.3	1.5	0	3.9 ×
	1275	91.5	8.1	0.4	0	
Temperature management in chilled facilities	341	91.8	5.9	2.4	0	3.9 ×
	1314	93.8	5.6	0.6	0	
Maintenance of facilities and structures	414	63.8	31.4	4.8	0	3.1 ×
	1674	79.8	18.7	1.6	0	
Maintenance of fixtures, equipment, water equipment and utensils	389	73.3	24.2	2.6	0	3.1 ×
	1465	86.5	12.7	0.8	0	
Temperature management in food production processes	283	86.2	10.3	3.5	0	3.0 ×
	924	91.1	7.7	1.2	0	
Sampling and own-check tests	188	81.4	13.3	5.3	0	2.2 ×
	1084	83.7	13.9	2.3	0.1	

* A = complies with requirements; B = minor issues that do not impair food safety or mislead consumers; C = issues that impair food safety or mislead consumers; D = major issues that considerably impair food safety or mislead consumers.

**Inspection items with more than 1000 inspections included.

### Association between inspection interval and inspection grades

#### Overall grades

When all the establishments were pooled together, there was a 0.14 (*p* = 0.02) difference in standard score estimates between inspections that resulted in grade A and inspections that resulted in grade C or D (Table 3). This means that on average, inspections that resulted in grade C or D had 5.9% longer inspection intervals. When analyses were conducted separately for different types of establishments, differences between inspections resulting in grade A and inspections resulting in grade C or D were seen in the fish industry, where the difference in standard score estimates was 0.28 (*p* = 0.01). To quantify this more accessibly, there was an 11.8% difference in the inspection intervals. In fish establishments, a difference in inspection intervals was also seen between inspections with grade A compared to inspections with grade B. The difference in standard score estimates was 0.16 (*p* = 0.01), meaning that inspections resulting in grade B had 7.0% longer inspection intervals.

**Table 3:** Standard score estimates for pre-planned inspections resulting in grades A, B, and C or D.

Establishment type	Standard score estimate		*p*-value	Average difference in inspection intervals (%)*	Standard score estimate		*p*-value	Average difference in inspection intervals (%)*	Standard score estimate		*p*-value	Average difference in inspection intervals (%)*
	Inspections graded A	Inspections graded B			Inspections graded A	Inspections graded C/D			Inspections graded B	Inspections graded C/D		
All establishments	− 0.07	− 0.02	0.13	2.1	− 0.07	0.07	0.02	5.9	− 0.02	0.07	0.12	3.8
Meat establishments	− 0.02	− 0.06	0.49	− 1.7	− 0.02	0.04	0.48	2.7	− 0.06	0.04	0.21	4.4
Fish establishments	− 0.16	0.00	0.01	7.0	− 0.16	0.12	0.01	11.8	0.00	0.12	0.24	4.9
Dairy establishments**												
Previous inspection A	− 0.11	0.01	0.18	5.0	− 0.11	0.38	0.08	21.0	0.01	0.38	0.18	16.0
Previous inspection B	− 0.04	− 0.06	0.85	− 1.1	− 0.04	− 0.33	0.19	− 12.3	− 0.06	− 0.33	0.21	− 11.3

Estimates were calculated using generalized estimating equations. A higher standard score estimate denotes a longer inspection interval.

*Calculated as difference between standard score estimates multiplied by mean standard deviation divided by mean inspection interval.

**Interaction between inspection grade and grade of the previous inspection; results interpreted separately based on grade of the previous inspection.

No statistically significant differences were seen in meat establishments. In dairy establishments, an interaction between the inspection grade and the grade of the previous inspection was observed. Thus, analyses were conducted separately based on the grade of the previous inspection. If the previous grade had been A, a longer inspection interval was associated with an inferior grade. If the grade of the previous inspection had been B, the results were the opposite. However, these findings were not statistically significant.

#### Inspection-item-specific grades

Eight inspection items displayed statistically significant associations between inspection interval and inspection grades (Table 4). In all of these, longer inspection intervals were associated with inferior inspection grades. Differences in inspection intervals were most frequently observed between inspections that resulted in grade A and those that resulted in grade C or D. These differences were also the widest; there was a 26.8% difference in the inspection interval for the inspection item “Own-check of listeria” and 23.3% difference for “Own check-testing of water and ice”, 16.8% difference for “Cleanliness and order of facilities and structures”, and 16.1% differences for the inspection item “Sampling and own-check tests”.

**Table 4:** Standard score estimates for inspection items at pre-planned inspections resulting in grades A, B, and C or D.

Inspection item**	Standard score estimate		*p*-value	Average difference in inspection intervals (%)*	Standard score estimate		*p*-value	Average difference in inspection intervals (%)*	Standard score estimate		*p*-value	Average difference in inspection intervals (%)*
	Inspections graded A	Inspections graded B			Inspections graded A	Inspections graded C/D			Inspections graded B	Inspections graded C/D		
Maintenance of facilities and structures (*n* = 2135)	0.00	0.04	0.12	2.2	0.00	0.28	0.004	14.5	0.04	0.28	0.03	12.3
Maintenance of fixtures, equipment, water equipment and utensils (*n* = 1827)	0.00	0.09	0.01	5.2	0.00	0.19	0.09	10.4	0.09	0.19	0.45	5.3
Cleanliness and order of facilities and structures (*n* = 3382)	0.00	0.06	0.07	2.7	0.00	0.35	0.001	16.8	0.06	0.35	0.01	14.1
Cleanliness of surfaces, fixtures, equipment and utensils (*n* = 3032)	0.00	0.04	0.30	2.0	0.00	0.29	0.02	14.1	0.04	0.29	0.06	12.1
Working hygiene of personnel (*n* = 1920)	0.00	0.06	0.19	3.7	0.00	0.02	0.85	1.0	0.06	0.02	0.69	− 2.7
Working clothes and protective clothes of personnel (*n* = 2213)	0.00	0.01	0.83	0.7	0.00	0.35	0.29	19.9	0.01	0.35	0.33	19.2
General hygiene of food production (*n* = 1070)	− 0.02	0.17	0.01	10.6	− 0.02	− 0.05	0.89	− 1.5	0.17	− 0.05	0.29	− 12.1
Separation of activities requiring different hygiene levels (*n* = 1168)	− 0.01	0.08	0.14	4.7	− 0.01	0.06	0.63	3.7	0.08	0.06	0.91	− 1.0
Hygiene of water supply points and equipment using water (*n* = 1596)	0.00	0.03	0.56	1.4	0.00	0.31	0.0501	16.9	0.03	0.31	0.10	15.5
Hygiene in storage and warehousing of foodstuffs (*n* = 1592)	0.00	0.05	0.22	2.9	0.00	0.19	0.18	10.5	0.05	0.19	0.41	7.6
Temperature management in chilled facilities (*n* = 1599)	− 0.01	0.13	0.11	6.8	− 0.01	0.23	0.23	11.6	0.13	0.23	0.67	4.8
Temperature management in food production processes (*n* = 1053)	0.00	0.00	0.99	0.0	0.00	0.23	0.18	12.7	0.00	0.23	0.25	12.7
Sampling and own − check tests (*n* = 1022)	0.00	0.08	0.23	4.1	0.00	0.31	0.01	16.1	0.08	0.31	0.12	12.0
Own − check testing of water and ice (*n* = 698)	0.00	0.18	0.03	9.2	0.00	0.45	< 0.0001	23.3	0.18	0.45	0.10	14.1
Own − check of listeria (*n* = 599)	0.00	0.11	0.24	5.2	0.00	0.55	0.002	26.8	0.11	0.55	0.054	21.5
Display of the inspection report (*n* = 1255)	0.00	0.06	0.44	3.5	0.00	0.00	0.99	0.2	0.06	0.00	0.85	− 3.3

Estimates were calculated using generalized estimating equations. A higher standard score estimate denotes a longer inspection interval.

*Calculated as difference between standard score estimates multiplied by mean standard deviation divided by mean inspection interval.

**Inspection items with *n* > 1000 included. In addition, inspection items with statistically significant findings and 500 > *n* > 1000 included.

## Discussion

In order to be able to develop food control, multifaceted research is necessary. In this study we found associations between inspection results and (1) inspection history, (2) inspection interval, and (3) pre-announcement. These findings are applicable when future directions regarding food control are established. For example, inspection history could be used in a more dynamically manner when deciding the timing of the next inspection.

Our results demonstrate that previous non-compliances can predict future violations of food safety legislation. A markedly greater proportion of inspections resulted in grades C or D if a previous inspection had resulted in grade B compared to grade A. This is consistent with a recent study concerning restaurant inspections in which 6.2% of inspections resulting in grade A were followed by grade C or D, whereas corresponding number was 17.4% if previous inspection had resulted in grade B^5^. Moreover, if an overall inspection grade was B, the number of individual inspection items graded B was associated with inferior grades at the next inspection. This can partly be explained by the fact that non-compliances more often go undetected when multiple non-compliances have already been detected^21^. These findings are noteworthy when determining the time of the next inspection. The fact that inspection history predicts inspection results could be emphasized more when determining inspection frequency. In Finland, current guidelines state that if six previous inspections have resulted in an excellent (A) overall grade, the inspection frequency could be decreased by up to 50%^4^. This is a rather rigid way of applying risk-based food control. This study shows that the four-level inspection scale used in Finland could be used in the implementation of a more dynamic approach in the determination of inspection interval. For example, inspection interval could be shortened if there are several inspection items graded B compared to just one. Moreover, a more dynamic approach could also consider the types of violations by using weighted grades depending on the risk of the violation as suggested by Lee et al.^22^ and Da Cunha et al.^23^.

Correction of non-compliances seems to be highly dependent on the type of violation, as the correction percentage (improvement from grades C or D to grades A or B) varied from 51 to 92% depending on the inspection item. Some of the inspection items were recognized as being exceptionally problematic for FBOs because (1) grade B was often followed by grade C or D, and (2) there often seemed to be problems in the correction of grade C or D. These inspection items included, for example, “Approval of facilities, structures and equipment”, “Sampling and own-check tests”, and “Maintenance of facilities and structures”. These are items that should be targeted more at inspections. Interestingly, adequacy and suitability of facilities, their maintenance, and record-keeping of own-check plan have been found to be topics that FBOs and inspectors most commonly disagree over^24^. Disagreement probably arises from the fact that making these corrections can be expensive, which likely decreases FBOs’ willingness to execute them.

Furthermore, our results indicate that longer inspection intervals are associated with inferior inspection grades. This may partly be explained by the hypothesis that if the inspection interval is extended, FBOs pay less attention to inspection items, which could result in a decrease in inspection grades. Thus, inspection intervals should not be overly prolonged. It is particularly alarming that inspections resulting in grades C or D, which indicate impaired food safety, were associated with longer inspection intervals. This finding should not be neglected in future guidelines on food control. On average, the inspection interval for those inspections was 5.9% longer than inspection intervals for inspections resulting in grade A. We hypothesize that this difference could be greater but is decreased because inspectors often recognize establishments that are in danger of slipping into more serious non-compliances, and thus inspect them earlier than usual, which leads to a shorter average inspection interval. This is supported by the previous finding by Läikkö-Roto et al.^11^ that 96.1% of officials stated that previous inspection findings had at least a “fairly much” effect on future inspections. They also noted that if FBOs failed to correct non-compliances, inspectors increased the inspection frequency. More frequent inspections are also favorable as they give the inspector a better understanding of the processes and problem areas in production, which enables better legislative guidance and helps FBOs to see the relevance of non-compliances in food safety, positively affecting FBOs’ attitudes toward food control^12^. This can also lead to more positive food safety climate which has been shown to reduce non-compliances^25^.

Our findings regarding the inspection interval and individual inspection items were consistent and showed that in many inspection items, a longer inspection interval leads to grades C or D more often. Inspection items in which an association was seen were mostly related to maintenance, cleanliness, and own-check tests. Maintenance and cleanliness are inspection items for which it is easy to see that a prolonged inspection interval could lead to non-compliances. Facilities wear out with time and some FBOs become accustomed to minor non-compliances, which become more serious over time if regular food control inspections are not carried out. Worn surfaces are also harder to clean properly. However, the question of why the inspection interval had such an effect on inspection grades of items related to own-check testing is more complicated. Own-check testing should be carried out routinely based on an own-check plan, but apparently it is commonly disregarded if not inspected regularly. One possible reason for this is that own-check testing is expensive and thus disregarded if regular reminders are not given. Hence, more emphasis should be targeted to the inspection of maintenance, cleanliness, and own-check testing.

Another factor affecting inspection grades is the pre-announcement of inspections. Pre-announced inspections were 1.3 times more likely to be graded A than unannounced, and unannounced were 2.4 times more likely to be graded C or D than pre-announced. As could be expected, easy-to-correct inspection items were the ones that announcements had the most effect on. These inspection items were related, for example, to working hygiene and cleanliness. This is in line with previous knowledge gained from restaurant studies. The effect of pre-announcements has been seen in restaurants in easy-to-correct categories such as personal hygiene and equipment cleanliness^14^. In a recent study, Kaskela et al.^5^ found that non-compliances were at least twice as common in unannounced inspections for most of the inspection items. They also concluded that differences were particularly wide in easy-to-correct inspection items. However, some inspection items for which we observed differences between pre-announced and unannounced inspections were not so self-evident. For example, there were many more non-compliances in the “Separation of activities requiring different hygiene level” and “Temperature management in food production processes” in unannounced inspections. We hypothesize that at least some of the FBOs also recognize those non-compliances, but either ignore them for financial reasons or do not fully understand the food safety risks entailed by them. Such FBOs can be described as calculative non-compliers or doubting compliers, respectively^26^. It would be beneficial for inspectors to differentiate between these so that proper control measures could be taken.

In our study only 22.0% of inspections were unannounced, which does not appear to be in accordance with the legislative goal of carrying out most of the inspections unannounced^2^. It is clear that pre-announcement can influence the inspection outcome as it gives FBOs the opportunity to prepare for the inspection, which can lead to inspection results that do not reflect the true situation. However, pre-announcement also has benefits as it promotes active managerial control^13^. The effect of the time from pre-announcement to inspection in food service establishments has been studied by Nwako^27^. It was found that notification one day before the inspection affected inspection grades, but notification one hour before the inspection did not give FBOs enough time to correct non-compliances but did give management time to logistically prepare for the inspection. Our results indicate that pre-announcement clearly affects inspection results in food production establishments. If an inspection is to be pre-announced, it should be considered to make the announcement as close to the inspection time as possible.

In our study, overall compliance was fairly high, as 91.1% of pre-planned inspections resulted in either an excellent (A) or good (B) grade. However, there were considerable differences between food establishment types. Inspection grades were better in dairy than in meat and fish establishments. These results are consistent with an earlier study in which meat and fish establishments subjected to enforcement measures were shown to have multiple non-compliances, whereas dairy establishments had only a few^28^. These differences between establishment types could be caused by how food safety risks are perceived. FBOs’ risk perception has been found to explain the occurrence of non-compliances^24^. In dairy establishments, risks are seen to be recognized and handled properly as there are only a few percent that do not perceive that there are food safety risks in their production, whereas in fish establishments the respective number was 14.8%, and for small-scale meat establishments slightly lower than that of fish establishments^12^. Another explanation for a greater non-compliance rate in meat establishments could be that there are more inspection items specific to meat establishments compared to fish and dairy establishments. Meat establishments have 23 specific inspection items, whereas fish and dairy establishments have six and two items, respectively^16^. As food control should be risk-based, these findings need to be considered when targeting the food control of food production establishments. We suggest that food control should give more attention to meat and fish establishments to improve compliance. An increase of inspection frequency could lead to improvements, but also the improvement of FBOs understanding in food safety risks would be important^24^. This could be achieved, for example, by increasing the advice given by inspectors during inspections.

This study has some limitations that need to be addressed. We were not able to investigate the association between inspection interval and correction of non-compliances at follow-up inspections. This is due to the complicated nature of inspection intervals following non-compliances. In this case, the inspection interval is dependent on the severity of the non-compliance and the time limit set to correct it. In order to study this, an experimental study design would be needed. Another limitation of this study was that it was not possible to statistically test differences between announced and unannounced inspections, and the stability and changes in the grades of individual inspection items. This is because repeated inspections at establishments set limitations on the statistical methodology that can be used. However, we perceive that for the most part the differences are so large that conclusions can be drawn based on crude numbers.

## Conclusions

The results of this study can be used in the development of risk-based food control. Currently, the majority of inspections at food production establishments are conducted pre-announced. Our results demonstrate that there is a difference in the grades between pre-announced and unannounced inspections. More inspections should be conducted unannounced when possible to capture the true situation in the establishment. Furthermore, our results indicate that minor non-compliances frequently precede more severe non-compliances, and that a prolonged inspection interval leads to an increase in the number of non-compliances. Thus, inspection history should be taken into account more comprehensively when determining the inspection frequency for an establishment. A more sophisticated approach to determining the inspection interval, based on the inspection history, could be adopted in Finland.

## Supplementary Information

Supplementary Information.

## Data Availability

The inspection report data that support the findings of this study can be requested from the Finnish Food Authority. Researchers are not able to provide data publicly available due to confidentiality agreement.
