# Supplementary material for: Factors affecting effectiveness of food control inspections in food production establishments in Finland
Source: Sci Rep. 2022 Mar 10;12:4230. doi: 10.1038/s41598-022-08204-1 (PMC8913778; doi:10.1038/s41598-022-08204-1)
Supplement: Supplementary file 1 — Supplementary Information. [file 41598_2022_8204_MOESM1_ESM.pdf]

Supplementary information

Factors affecting effectiveness of food control inspections in food production establishments in Finland

Mikko Kosola[1], Katri Kiviniemi[1], Janne Lundén[1]
[1]Department of Food Hygiene and Environmental Health, Faculty of Veterinary Medicine, P.O. Box 66, FI-00014 University of Helsinki, Finland

Supplementary table S1. Grade distribution of individual inspection items grouped by the previous grade of the item. Inspection items with at least 500 observations included.

			Grade of the inspection item			
**Inspection item**	**Previous grade of the inspection item**	**n**	**A**	**B**	**C**	**D**
**Approval of facilities, structures and equipment**	A	538	95.9	3.0	0.7	0.4
	B	45	62.2	26.7	8.9	2.2
	C	18	66.7	5.6	22.2	5.6
	D	4	50.0	25.0	0.0	25.0
**Approval of activities**	A	561	98.0	0.9	0.9	0.2
	B	12	58.3	33.3	8.3	0.0
	C	11	63.4	27.3	9.1	0.0
	D	3	33.3	33.3	33.3	0.0
**Compliance of water intended for human consumption with requirements**	A	500	97.6	2.2	0.2	0.0
	B	18	77.8	16.7	5.6	0.0
	C	4	50.0	25.0	25.0	0.0
	D	1	100.0	0.0	0.0	0.0
**General compliance of own-check with requirements**	A	653	86.2	12.4	1.4	0.0
	B	218	43.6	49.1	7.3	0.0
	C	62	16.1	40.3	32.3	11.3
	D	6	16.7	0.0	33.3	50.0

**Maintenance of facilities and structures**	A	1656	86.0	13.2	0.7	0.1
	B	614	35.5	54.7	9.3	0.5
	C	97	22.7	47.4	24.7	5.2
	D	19	10.5	21.1	21.1	47.4
**Maintenance of fixtures, equipment, water equipment and utensils**	A	1596	87.8	11.2	1.0	0.0
	B	367	45.5	46.9	7.4	0.3
	C	57	35.1	36.8	26.3	1.8
	D	6	0.0	66.7	0.0	33.3
**Cleanliness and order of facilities and structures**	A	2763	87.3	11.8	0.9	0.0
	B	740	46.9	45.4	7.4	0.3
	C	119	27.7	44.5	23.5	4.2
	D	13	7.7	30.8	23.1	38.5
**Cleanliness of surfaces, fixtures, equipment and utensils**	A	2700	90.6	8.8	0.6	0.1
	B	453	56.1	37.8	5.7	0.4
	C	61	31.2	37.7	26.2	4.9
	D	7	57.1	14.3	28.6	0.0
**Vermin control**	A	785	92.5	7.0	0.5	0.0
	B	109	63.3	30.3	6.4	0.0
	C	20	35.0	35.0	20.0	10.0
	D	2	0.0	50.0	0.0	50.0
**Disposal of wastes and wastewater**	A	678	93.5	6.5	0.0	0.0
	B	83	61.5	34.9	3.6	0.0
	C	8	50.0	37.5	0.0	12.5
	D	1	0.0	100.0	0.0	0.0
**Working hygiene of personnel**	A	1805	91.6	7.8	0.6	0.0
	B	226	56.6	34.5	8.9	0.0
	C	45	40.0	37.8	15.6	6.7
	D	3	33.3	66.7	0.0	0.0

**Working clothes and protective clothing of personnel**	A	2154	94.6	5.2	0.3	0.0
	B	181	70.2	26.0	3.9	0.0
	C	27	37.0	33.3	22.2	7.4
	D	1	0.0	100.0	0.0	0.0
**Instruction, guidance and training of personnel**	A	432	94.9	4.9	0.2	0.0
	B	58	56.9	36.2	3.5	3.5
	C	11	63.6	18.2	18.2	0.0
	D	1	0.0	0.0	100.0	0.0
**Verification of hygiene proficiency**	A	498	94.4	5.0	0.6	0.0
	B	60	71.7	23.3	5.0	0.0
	C	15	46.7	26.7	26.7	0.0
	D	0	-	-	-	-
**General hygiene of food production**	A	1047	91.7	7.2	1.1	0.1
	B	134	61.2	33.6	4.5	0.8
	C	24	41.7	41.7	16.7	0.0
	D	2	0.0	50.0	50.0	0.0
**Separation of activities requiring different hygiene levels**	A	1107	90.9	8.0	1.1	0.0
	B	192	45.8	47.4	6.8	0.0
	C	32	56.3	31.3	9.4	3.1
	D	1	0.0	0.0	100.0	0.0
**Hygiene of water supply points and equipment using water**	A	1511	90.4	9.1	0.5	0.0
	B	224	62.1	32.6	5.4	0.0
	C	33	36.4	36.4	18.2	9.1
	D	3	33.3	33.3	0.0	33.3
**Hygiene in thawing, chilling and quick-freezing**	A	669	91.8	7.2	1.1	0.0
	B	75	65.3	28.0	6.7	0.0
	C	22	50.0	27.3	18.2	4.6

	D	1	0.0	100.0	0.0	0.0
**Hygiene in wrapping and packing**	A	922	93.5	6.2	0.3	0.0
	B	97	55.7	36.1	6.2	2.1
	C	12	66.7	16.7	8.3	8.3
	D	2	50.0	0.0	50.0	0.0
**Hygiene in storage and warehousing of foodstuffs**	A	1456	88.5	10.0	1.4	0.0
	B	272	56.3	36.4	7.0	0.4
	C	54	46.3	33.3	20.4	0.0
	D	2	100.0	0.0	0.0	0.0
**Hygiene in handling and storage of by-products**	A	412	86.4	12.1	1.5	0.0
	B	112	57.1	33.0	9.8	0.0
	C	23	30.4	43.5	21.7	4.4
	D	1	0.0	100.0	0.0	0.0
**Temperature management in chilled facilities**	A	1594	94.8	4.5	0.7	0.0
	B	160	71.3	23.1	5.6	0.0
	C	27	29.6	40.7	18.5	11.1
	D	2	0.0	50.0	50.0	0.0
**Temperature management in food production processes**	A	1028	92.4	6.2	1.4	0.0
	B	128	61.7	32.0	6.3	0.0
	C	35	68.6	25.7	5.7	0.0
	D	2	0.0	50.0	50.0	0.0
**General labelling**	A	433	74.1	22.2	3.5	0.2
	B	258	43.4	46.1	10.1	0.4
	C	65	44.6	29.2	24.6	1.5
	D	4	0.0	50.0	0.0	50.0
**Traceability of foodstuffs**	A	516	86.8	9.7	3.3	0.2
	B	90	66.7	24.4	7.8	1.1

	C	46	39.1	26.1	30.4	4.4
	D	6	16.7	16.7	50.0	16.7
**Sampling and own-check tests**	A	949	90.4	8.5	1.1	0.0
	B	195	47.2	41.0	11.8	0.0
	C	86	29.1	22.1	40.7	8.1
	D	4	25.0	25.0	25.0	25.0
**Own-check testing of water and ice**	A	701	89.2	9.0	1.9	0.0
	B	128	60.2	28.1	10.9	0.8
	C	41	51.2	19.5	22.0	7.3
	D	2	50.0	50.0	0.0	0.0
**Own-check of listeria**	A	598	91.1	8.0	0.8	0.0
	B	104	53.9	32.7	13.5	0.0
	C	51	41.2	27.5	25.5	5.9
	D	6	16.7	33.3	33.3	16.7
**Display of the inspection report**	A	1230	95.9	4.2	0.0	0.0
	B	120	55.8	35.0	9.2	0.0
	C	14	42.9	21.4	35.7	0.0
	D	0	-	-	-	-
